# Dysthyroid optic neuropathy: emerging treatment strategies

**DOI:** 10.1007/s40618-023-02036-0

**Published:** 2023-02-18

**Authors:** M. Pelewicz-Sowa, P. Miśkiewicz

**Affiliations:** Department of Internal Medicine and Endocrinology, Medical University of Warsaw, 02-091 Warsaw, Poland

**Keywords:** Dysthyroid optic neuropathy, Graves’ orbitopathy, Methylprednisolone, Teprotumumab, Tocilizumab, Rituximab

## Abstract

**Purpose:**

Dysthyroid optic neuropathy (DON) is a rare sight-threatening complication of Graves’ disease. First-line treatment for DON consists of high-dose intravenous methylprednisolone (ivMP), followed by immediate orbital decompression (OD) if the response is poor or absent as recommended by the 2021 European Group on Graves’ orbitopathy guidelines. The safety and efficacy of the proposed therapy have been proven. However, consensus regarding possible therapeutic options for patients with contraindications to ivMP/OD or resistant form of disease is missing. This paper aims to provide and summarize all available data regarding possible alternative treatment strategies for DON.

**Methods:**

A comprehensive literature search within an electronic database was performed including data published until December 2022.

**Results:**

Overall, 52 articles describing use of emerging therapeutic strategies for DON were identified. Collected evidence indicates that biologics, including teprotumumab and tocilizumab, may be considered as an important possible treatment option for DON patients. Rituximab should be avoided in DON due to conflicting data and risk of adverse events. Orbital radiotherapy could be beneficial for patients with restricted ocular motility classified as poor surgical candidates.

**Conclusion:**

Only a limited number of studies have been dedicated to the therapy of DON, mostly retrospective with a small sample size. Clear criteria regarding diagnosis and resolution of DON do not exist, which restricts comparison of therapeutic outcomes. Randomized clinical trials and comparison studies with long-term follow-ups are necessary to verify the safety and efficacy of each therapeutic option for DON.

## Introduction

Graves’ orbitopathy (GO) is the major extrathyroidal manifestation of Graves’ disease [1]. The pathogenesis of this autoimmune orbital disorder is based on inflammation, adipogenesis and excessive production of glycosaminoglycans, resulting in enlarged eye muscles and expansion of the orbital connective tissue [2]. It manifests with pain, diplopia, proptosis, redness and swelling of the eyelids, conjunctiva and caruncle [3, 4]. Treatment choice depends mostly on the clinical activity, severity and duration of GO.

Dysthyroid optic neuropathy (DON) is a sight-threatening complication which occurs in approximately 3–7% of patients with GO [5, 6]. Diagnosis of DON is made following clinical, ophthalmological and radiological examination. Decreased visual acuity (VA), impaired color vision, visual field defects found in clinical evaluation, as well as optic disk swelling/pallor and relative afferent pupillary defect (RAPD) revealed during ophthalmological examination are indicative of DON [7]. Magnetic resonance imaging and/or computed tomography is used to assess compression of the optic nerve exerted by swollen extraocular muscles within the orbital apex (apical crowding) and/or rarely to visualize optic nerve stretching. Differential diagnosis should exclude other orbital pathologies affecting visual functioning, such as glaucoma, cataract, orbital tumors, idiopathic orbital inflammation, orbital arteriovenous malformations, as well as rare disorders including IgG4-related ophthalmic disease and Erdheim–Chester disease [8–10]. However, clear criteria regarding diagnosis and resolution of DON have not been established.

First-line treatment for DON recommended by the 2021 European Group on Graves’ orbitopathy (EUGOGO) guidelines consist of high-dose intravenous methylprednisolone (ivMP) pulse therapy with 0.5–1.0 g given for 3 consecutive days or on every second day which may be repeated for another week. If the response is poor or absent, orbital decompression (OD) must be performed promptly within 1–2 weeks [11]. The described therapy is also advised in the 2022 Consensus Statement by the American Thyroid Association and European Thyroid Association [12].

So far, the majority of the published literature concerning DON provides information regarding the effectiveness of the combined approach with ivMP and decompression [13–21]. However, vision recovery is not always possible to achieve with the recommended therapy [16, 22, 23] and deterioration of the optic nerve function, including relapse of DON following completion of the basic treatment, has also been described [20, 24–26]. Moreover, the recommended therapy cannot be applied in all patients or must be discontinued due to various contraindications and side effects [17, 27–31], especially in those with preexisting comorbidities [32, 33].

Clinical deterioration of ophthalmic signs and symptoms in moderate-to-severe and active GO requires implementation of second-line treatment. But, when it comes to DON, alternative treatment strategies are missing and only a limited number of studies have been dedicated to the therapy of DON, mostly retrospective with a small sample size. Therefore, as optimal therapy must be applied immediately due to possible permanent dysfunction in the optic nerve caused by any delay, managing patients with DON still remains a major challenge.

In this paper, we present and review emerging alternative treatments for DON.

## Dysthyroid optic neuropathy: basic treatment

### Intravenous methylprednisolone and orbital decompression

First-line treatment for DON comprises of high-dose ivMP (0.5–1.0 g) pulses given for 3 consecutive days or on every second day, repeated if necessary, and followed by immediate OD in case of insufficient response or disease deterioration. Several studies have analyzed the effectiveness of ivMP therapy in various doses combined with OD [15, 17–20], but only four studies, including one prospective, have described the efficacy of the recommended DON therapy [13, 14, 16, 22]. As clear criteria of DON resolution have not been established, diverse indicators of DON improvement have been applied in the published literature, which restricts comparison of the therapeutic outcomes. However, as DON may lead to irreversible loss of vision, crucial treatment decisions depend mostly on improvement in VA.

Studies evaluating the recommended protocol described response (defined as VA 0.0–0.3 logMAR) rate to high-dose ivMP between 22 and 61%. Following OD, positive response increased to 67–87%. Additionally, randomized clinical trial (RCT) by Wakelkamp et al. [13] found that ivMP is a preferable treatment choice over OD. Surgery performed primarily did not result in a better outcome, and 83% patients required further ivMP pulses. In contrast, only 44% of patients treated first with ivMP needed subsequent OD.

Surgical decompression is conducted to directly reduce pressure within the orbital apex by extending the available orbital volume through orbital wall removal, which may be combined with fat excision in case of its hypertrophy. Inferomedial wall removal is generally considered the first approach in DON patients, as it reaches deeper into the apical complex where compression of the optic nerve is produced [34]. Several surgical approaches have been described, including transnasal, transcaruncular, transcutaneous and transantral. Recent studies show that transnasal endoscopic (TEOD) approach has become a widely used and efficient method when decompressing medial or inferior wall in patients with DON. It provides good visualization and access to the orbital apex, causing less damage to the muscles and ligaments without producing external scar [35–41]. Additionally, TEOD was described to result in significantly greater improvement in VA in DON patients compared to other popular approach, such as transcaruncular [42–44]. Severe cases may require additional lateral decompression which results in greater proptosis reduction [45–48].

According to Wakelkamp et al. [13], OD must be applied immediately in patients refractory to ivMP or with deterioration of clinical condition. However, direct indications regarding optimal timing for performing OD are missing. The World Health Organization defines mild vision impairment as VA > 0.3 logMAR, and moderate as VA > 0.5 logMAR. Additional factors such as older age, smoking, long-lasting disease, history of resistance to ivMP, poor initial VA, optic disk swelling, unstable thyroid function and high level of thyrotropin receptor antibodies have been described to be associated with potential need for immediate OD [14, 49]. Therefore, as rapid treatment decisions and personalized approach are required, patients should be referred to highly specialized centers with large surgical experience where combined endocrine, ophthalmological and surgical management is available.

## Dysthyroid optic neuropathy: alternative treatments

Diverse criteria of DON resolution were applied in the analyzed literature. Successful therapy was defined as disease stabilization with no need of further DON treatment with improvement in VA and/or color vision/visual field/RAPD.

### Additional intravenous methylprednisolone pulses

According to the 2021 EUGOGO guidelines, cumulative dose of ivMP in DON therapy should not exceed 8.0 g per cycle due to increased rate of adverse events [11]. However, the end point of the basic therapy with high-dose ivMP and OD has not been determined due to lack of specific criteria of DON resolution, and consensus regarding the optimal moment for performing OD is also missing. Signs of DON may persist even following the recommended therapy (clinical: decreased VA, reduced color vision, visual field defects; ophthalmological: swollen or pale optic disk, RAPD; radiological: apical crowding, optic nerve stretching).

Several studies have described the positive results obtained by applying additional ivMP pulses, both in patients with partial response to the first-line treatment [14, 16, 19] and in cases of persistent DON despite medical and surgical decompression [22]. A recently published study [50] found that applying additional ivMP pulses in a 12-week protocol (4.5 g or 7.5 g), once the DON treatment with high-dose ivMP with/without OD is completed, provides stabilization or further improvement of clinical outcome, including VA, color vision, clinical activity score (CAS) and proptosis. Further ivMP pulses may also impact quality of life of DON patients [51].

Neither of the reports described significant adverse events, although cumulative doses of ivMP exceeding 8.0 g were applied. However, as various studies proved ivMP to be associated with multiple side effects, thorough examination of patients, including assessment of aspartate and alanine aminotransferase serum levels, and blood pressure measurements, must be performed prior to administration of each pulse.

### Teprotumumab

The insulin-like growth factor 1 receptor (IGF-1R) plays a critical role in the expansion of the orbital tissues in patients with GO due to its overexpression in orbital fibroblasts and immune cells [52–54]. Teprotumumab, a human monoclonal antibody directed against IGF-1R, is the first FDA-approved treatment for active GO, administered intravenously every 3 weeks in eight infusions with an initial dose of 10 mg/kg followed by 20 mg/kg [55]. The 2021 EUGOGO guidelines suggest applying teprotumumab as second-line treatment for moderate-to-severe and active GO.

Although patients with DON were excluded from the phase 2 and 3 clinical trials [53, 55], studies demonstrated reduction in the extraocular muscle size, also within the orbital apex, as well as significant improvement in CAS, proptosis, diplopia and quality of life in patients with GO compared to placebo, making teprotumumab a potential treatment option for DON.

To date, eight reports with a total number of 20 DON patients (29 eyes) managed with teprotumumab have been published [56–63] (Tables 1 and 2).

**Table 1:** Summary of the analyzed studies regarding teprotumumab

Study	Design	Patients	Eyes	Previous treatment of DON with OP/ivMP /OD/ORT	Simultaneous treatment	Teprotumumab infusions^c^	Resolution of DON^c^-% of all eyes	FU^g^	Recurrence of DON during FU	OD after tocilizumab during FU
Cheng et al. [57]	Case report	1	1	OP, ivMP, OD	–	8	100%	51	Yes (after 51 weeks)	Yes (after 51 weeks)
Lopez et al. [59]	Case report	1	2	OP	OP^b^	8	100%	24	No	No
Sears et al. [58]	Case report	1	1	ivMP ORT	–	8	100%	21	No	No
Hwang et al. [56]	Case report	1	2	OP	–	8	100%	21	No	No
Slentz et al. [60]	Case report	1	1	–	–	8	100%	25	No	No
Sears et al. [63]	Case series	10	17	5^a^ OP 9^a^ ivMP 5^a^ OD 2^a^ ORT	–	8	64.7%^f^	121–54	No	No
Diniz et al. [62]	Case series	3	3	3^a^ ivMP 2^a^ OD	–	3/3/6^d^	100%	9–19	No	No
Chiou et al. [61]	Case series	2	2	2^a^ ivMP	–	2/3^d^	100%	7–9	No	No

*DON* dysthyroid optic neuropathy, *OP* oral prednisolone, *ivMP* intravenous methylprednisolone, *OD* orbital decompression, *ORT* orbital radiotherapy, *FU* follow-up, *VA* visual acuity, *RAPD* relative afferent pupillary defect

^a^Number of patients receiving treatment

^b^OP with the first three infusions of teprotumumab

^c^Number of infusions (initial dose of 10 mg/kg, followed by 20 mg/kg intravenously)

^d^Number of infusions each patient received

^e^Defined as stabilization of disease with no need of further DON treatment and improvement in VA and/or color vision/visual field/RAPD

^f^7 patients and 11 eyes

^g^FU in weeks

**Table 2:** Clinical features of eyes with DON before and after treatment with teprotumumab

Study	Before teprotumumab			After teprotumumab		
	VA^a^	Proptosis^b,c^	Additional findings^c^	VA^a^	Proptosis^b,c^	Additional findings^c,d,e^
Cheng et al. [57]	0.6	20.5	Impaired CV	0.0	15.5	Restoration of CV
Lopez et al. [59]	0.3	25	Impaired CV	0.1	18	Restoration of CV
	0.4	27	Impaired CV	0.0	21	Restoration of CV
Sears et al. [58]	0.8	23	Impaired CV	0.2	19	Restoration of CV
Hwang et al. [56]	0.7	20.5	Impaired CV	0.3	15.5	Restoration of CV
	HM	20.5	Impaired CV	0.7	16.0	Improvement in CV
Slentz et al. [60]	0.1	25	VFD	0.0	20	Restoration of VF
Chiou et al. [61]	0.1	26.5	RAPD VFD Red color desaturation	0.1	23.5	Resolution of RAPD Restoration of CV and VF
	0.1	20	RAPD VFD	0.1	15	Resolution of RAPD Restoration of VF
Diniz et al. [62]	1.2	Missing	RAPD Impaired CV	0.0	Missing	Resolution of RAPD Restoration of CV
	0.3	Missing	RAPD Impaired CV VFD	0.5	Missing	Resolution of RAPD Improvement in CV and VF
	1.0	Missing	RAPD Impaired CV VFD	0.4	Missing	Resolution of RAPD Restoration of CV Improvement in VF
Sears et al. [63]	HM	30	Impaired CV	1.6	27	Impaired CV
	HM	30	Impaired CV	1.6	28	Impaired CV
	HM	28	Impaired CV	1.6	25	Impaired CV
	HM	29	Impaired CV	1.6	26	Impaired CV
	1.0	30	Impaired CV	0.4	26	Restoration of CV
	1.0	29	Impaired CV	0.2	24	Restoration of CV
	0.2	Missing	Impaired CV	0.0	Missing	Restoration of CV
	0.3	Missing	VFD	0.1	Missing	Missing
	0.8	Missing	RAPD Impaired CV	0.1	Missing	Resolution of RAPD Restoration of CV
	0.7	Missing	RAPD Impaired CV	0.1	Missing	Resolution of RAPD Restoration of CV
	0.1	Missing	RAPD	0.0	Missing	Resolution of RAPD
	0.7	30	Impaired CV	0.1	29	Restoration of CV
	0.5	30	RAPD Impaired CV	0.3	29	Resolution of RAPD Restoration of CV
	0.2	27	Missing	0.0	22	Missing
	0.3	26	RAPD	0.0	23	Resolution of RAPD
	0.7	Missing	Impaired CV	0.5	Missing	Impaired CV
	HM	Missing	RAPD	1.0	Missing	RAPD

*DON* dysthyroid optic neuropathy, *VA* visual acuity, *HM* hand motion, *CV* color vision, *VFD* visual field defect, *VF* visual field, *RAPD* relative afferent pupillary defect, *logMAR* logarithm of the minimum angle of resolution

^a^VA presented as logMAR

^b^Proptosis expressed in mm

^c^Missing indicates that authors of the study did not provide the information

^d^Restoration expresses full recovery

^e^Improvement expresses partial recovery

Teprotumumab was successful in 17 individuals (23 eyes). In 16 of them (22 eyes), teprotumumab was used as an alternative treatment. Previous therapy with ivMP with or without OD/orbital radiotherapy (ORT) failed or had to be discontinued due to intolerance. Three patients (6 eyes) resistant to teprotumumab had long-lasting DON (over 12 months) and were previously treated with ivMP, two of them also with OD.

The largest case series mentioned above [63] included 10 patients with DON (17 eyes) resistant to previous therapy. Teprotumumab (8 infusions; 10 mg/kg first infusion; 20 mg/kg for subsequent infusions) applied as alternative treatment resulted in significant improvement in VA, CAS and proptosis. Most patients experienced early response within two infusions. This rapid improvement is consistent with the clinical trials [53, 55].

### Tocilizumab

Interleukin-6 (IL-6) is a pro-inflammatory cytokine found in higher concentrations in patients with GO. Through T and B cell activation, IL-6 induces adipogenesis and synthesis of glycosaminoglycans promoting expansion of the orbital volume [64–66]. Tocilizumab (TCZ) is a recombinant humanized monoclonal antibody directed against the IL-6 receptor, which, according to the 2021 EUGOGO guidelines, may be considered as second-line treatment for moderate-to-severe and active GO [11].

TCZ (8 mg/kg in 4 monthly infusions) was found in RCT [67] to significantly reduce CAS and median proptosis values in patients with moderate-to-severe and active GO refractory to ivMP compared to placebo. Although generally well tolerated, a higher rate of infections and headaches was noted in patients treated with TCZ. Improvement in CAS, proptosis and diplopia following TCZ infusions was also described in a few retrospective studies [68–70]. One of the papers noted significant improvement in VA following first month of TCZ treatment [70]. These findings highlight the potential utility of TCZ in DON patients.

Until now, seven reports with a total number of 14 DON patients (16 eyes) treated with TCZ have been published (Tables 3 and 4) [70–76].

**Table 3:** Summary of the analyzed studies regarding tocilizumab

Study	Design	Patients	Eyes	Previous treatment of DON with ivMP /OD/ORT	Simultaneous treatment	Tocilizumab infusions^a^	Resolution of DON^c^-% of all eyes	FU in weeks	Recurrence of DON during FU	OD after Tocilizumab during FU
Mehmet et al. [72]	Case report	1	2	ivMP, ORT	–	4	100%	12	No	No
Kaplan et al. [76]	Case report	1	1	ivMP, OD, ORT	–	11	100%	84	Yes^e^	No
Pascual-Camps et al. [71]	Case report	1	2	–	–	4	100%	64	No	No
Maldiney et al. [75]	Case report	1	1	ivMP	ivMP	8	100%	28	No	No
Rodríguez et al. [74]	Case report	1	1	ivMP, OD	–	6	100%	16	No	No
Sy et al. [73]	Case series	2	2	ivMP, OD	–	4/4^b^	100%	12	No	No
Sánchez-Bilbao et al. [70]	Case series	7	7	ivMP, OD	Missing	Missing	Improvement in VA insignificant	65^d^	Missing	Missing

*DON* dysthyroid optic neuropathy, *ivMP* intravenous methylprednisolone, *OD* orbital decompression, *ORT* orbital radiotherapy, *FU* follow-up, *VA* visual acuity, *RAPD* relative afferent pupillary defect

^a^Number of infusions (8 mg/kg every 4 weeks, intravenously)

^b^Number of infusions each patient received

^c^Defined as stabilization of disease with no need of further DON treatment and improvement in VA and/or color vision/visual field/RAPD

^d^Mean time

^e^Recurrence of DON after 10 months following the first six infusions of tocilizumab; patient received five additional infusions obtaining again resolution of DON

**Table 4:** Clinical features of eyes with DON before and after treatment with tocilizumab

Study	Before tocilizumab		After tocilizumab	
	VA^a^	Additional findings^b^	VA^a^	Additional findings^b,c^
Mehmet et al. [72]	0.5	VFD	0.2	Improvement in VF
	0.4	VFD	0.0	Improvement in VF
Sy et al. [73]	HM	Missing	0.3	Missing
	HM	Missing	0.4	Missing
Pascual-Camps et al. [71]	1.0	VFD	0.0	Improvement in VF
	1.0	VFD	0.0	Improvement in VF
Rodríguez et al. [74]	1.3	Missing	0.0	Missing

*DON* dysthyroid optic neuropathy, *VA* visual acuity, *HM* hand motion, *VFD* visual field defect, *VF* visual field, *RAPD* relative afferent pupillary defect, *logMAR* logarithm of the minimum angle of resolution

^a^VA presented as logMAR

^b^Missing indicates that authors of the study did not provide the information

^c^Improvement expresses partial recovery

TCZ (4–8 infusions) was successful in 6 patients (8 eyes). In five of them (6 eyes), TCZ was applied as alternative treatment following unsuccessful therapy with ivMP with or without OD/ORT [71–75]. One patient (2 eyes) received TCZ as first-line treatment with no previous use of glucocorticoids (GCs) [71].

Alternative treatment with TCZ was insufficient in 8 patients (8 eyes) resistant to ivMP and OD. A group of seven of them (7 eyes) was analyzed together in one retrospective study which found no significant improvement in VA after 1 year of treatment with TCZ [70, 76].

### Rituximab

Rituximab (RTX) is a chimeric mouse–human monoclonal antibody directed against the CD20 antigen on B lymphocytes resulting in depletion of circulating B lymphocytes and transient (4–6 months) immunosuppression [77]. The 2021 EUGOGO guidelines [11] suggest considering RTX as second-line treatment for moderate-to-severe and active GO excluding patients with potential risk of developing DON. Although current recommendations do not address the use of RTX in DON patients, previous guidelines clearly advised against its administration due to conflicting data and risk of adverse events [78].

To date, four reports (overall 43 patients) described the occurrence of DON in 8 patients (18.6%; number of eyes missing) with moderate-to-severe and active GO following RTX infusions in various doses [79–82]. Additionally, two studies (overall 32 patients) evaluating the efficacy of RTX in moderate-to-severe and active GO observed 3 cases (9.1%) of severe cytokine release syndrome causing vision impairment with marked periorbital edema [81, 83].

Nevertheless, due to promising results in moderate-to-severe and active GO [83, 84] and despite potential adverse events, six reports describing 13 DON patients (number of eyes missing) treated with RTX have been published [83, 85–89].

In 11 DON cases, alternative combination treatment with RTX (various doses: 0.01–2.0 g) and GCs/ORT/OD was successful. Previous therapy with ivMP with/without OD failed [83, 87–89]. RTX (2 × 1.0 g) was insufficient in 2 cases, either as first-line treatment for DON or following ivMP pulses (3.0 g). Although transient (2–4 months) improvement in VA was achieved following RTX, further OD was required due to recurrence of DON with deterioration of vision [85, 86].

### Mycophenolate mofetil

Mycophenolate mofetil (MMF) is an inosine monophosphate dehydrogenase inhibitor, which suppresses proliferation of B and T lymphocytes, decreasing the production of antibodies. MMF induces apoptosis of activated T lymphocytes, reducing chemotaxis and immune cell recruitment [90]. Moreover, it disturbs fibroblast function and proliferation [91, 92]. The 2021 EUGOGO guidelines [11] decided to include MMF into the first-line treatment for moderate-to-severe and active GO in combination with ivMP, as it was found to result in significantly greater improvement in CAS and orbital signs and symptoms compared to ivMP alone [93, 94]. This highlights the potential utility of including MMF into the basic DON treatment, but consensus regarding the use of MMF in DON is missing.

However, two reports (overall 136 patients) [94, 95] evaluating the safety and efficacy of MMF (0.72 g daily for 24 weeks) in combination with ivMP pulses (1.5–4.5 g) in patients with moderate-to-severe and active GO observed occurrence of DON in 12 patients (8.8%; number of eyes missing) during the treatment.

So far, only one study [96] evaluated the effects of additional treatment with MMF in ten DON patients previously treated with ivMP with or without OD/ORT (various doses). MMF was applied during or directly after ivMP for a median time of 76 weeks. Significant improvement was observed only in CAS. Overall, three patients experienced relapse of DON within 78 weeks from baseline.

### Orbital radiotherapy

Ionizing radiation induces cell death by breaking double-stranded DNA [97]. Radiotherapy may interfere with the natural course of GO through apoptosis and disruption in functioning of orbital fibroblasts, macrophages and lymphocytes, which play a crucial role in the pathophysiology of the disease [2]. The 2021 EUGOGO guidelines recommend a combination of ORT with GCs as second-line treatment for moderate-to-severe and active GO, especially in patients with reduced eye muscle motility, excluding those with diabetic or hypertensive retinopathy and younger than 35 years [11]. According to ATA, including ORT into DON treatment may be considered [12]. Nevertheless, clear consensus regarding the use of ORT in DON patients is missing.

Several retrospective studies described the use of radiotherapy in DON patients, but they are mostly limited to a small sample size with distinct concurrent treatments, usually with oral GCs and OD [98–112]. So far, only one study described the use of ORT in combination with ivMP (20 Gy and 4.5 g) in a total number of 9 DON patients (number of eyes missing) obtaining resolution of DON in all cases with no recurrence during the 12-month follow-up and no major side effects [113].

The largest case series (104 patients, 163 eyes) described resolution of DON, defined as no need of urgent OD during acute phase of the disease in 98 patients (94.2%) and 152 eyes (93.3%) treated with oral GCs and ORT (20 Gy) as first-line treatment. Significant improvement in VA, color vision, visual field and RAPD was observed. Nevertheless, the retrospective character of the study with a long period of patient recruitment (22 years) should be considered while evaluating the results [98].

Moreover, ORT in addition to ivMP (20 Gy and 4.5 g) was described by Shams et al. [114] to prevent occurrence of DON in moderate-to-severe and active GO. None of the 91 patients developed DON, following combined therapy compared to 25 (17%) patients receiving ivMP alone (after average time of 3.2 years).

In contrast, ORT may also cause transient swelling of the orbital connective tissue, which may result in deterioration of DON despite simultaneous administration of ivMP (6 Gy and 3 g) as described by Hersh et al. [108].

### Limitations of the study

1. Clear criteria of DON recognition and resolution do not exist, which restricts comparison of the therapeutic outcomes.
2. Data evaluating management of DON is scarce and comprised mostly of retrospective studies, small case series, case reports and only a few RCTs.
3. Analyzed reports apply various criteria of DON resolution and provide limited information regarding follow-up results.

## Conclusions

1. High-dose ivMP, followed by OD remains the treatment of choice in DON as described in the 2021 EUGOGO guidelines.
2. Biologics, including FDA-approved teprotumumab and TCZ, may be considered as an important treatment option for DON.
3. ORT can be considered in DON patients classified as poor surgical candidates with restricted ocular motility.
4. RTX should be avoided in patients with DON due to conflicting data and risk of adverse events.
5. Consensus regarding alternative treatment strategies for DON is missing and its establishment is highly required. Further RCTs and comparison studies with long-term follow-ups are necessary to evaluate the safety and effectiveness of each therapeutic option in the treatment of DON.

## Data Availability

The datasets generated during and/or analysed during the current study are available from the corresponding author on reasonable request.
